# Basic principles of prenatal screening for aneuploidies

**DOI:** 10.1186/1755-8166-7-S1-I15

**Published:** 2014-01-21

**Authors:** Prakash Gambhir

**Affiliations:** 1 Birthright Genetic Clinic, Pune, India

## 

Prenatal screening processes are important public health interventions to counter common genetic disorders. For India prenatal screening against neural tube defects, thalassemia and Down syndrome is relevant to the health needs. However, there are many misconceptions regarding the principles of screening. Many clinicians wrongly consider it as substitute for prenatal diagnosis. Basically, prenatal screening protocols can be applied on a wide scale to identify women at risk of bearing a child with the common genetic disorder in that population.

Prenatal screening for Down syndrome and other chromosomal abnormalities is a rapidly evolving science. It initially started with offering prenatal diagnosis to elderly mothers. The identification of low maternal serum alpha-fetoprotein levels as a marker in Down syndrome led to quest and recognition of many serum markers and maternal serum markers of AFP uE3 and hCG were used in combination as triple test and with addition of Inhibin as the quadruple test. The identification of Nuchal translucency as a marker in 1^st^ trimester led to much desired early risk prediction along with PaPPA and free beta hCG. It was also found that this test led to early risk determination and an early prenatal diagnosis with significantly better detection rate of 85 to 90%. This detection rate is head and shoulders better than the detection rate for the triple test rate of 65 to 70%.

It has also been noted that combination of these markers can also lead to risk prediction of other chromosomal abnormalities. Presently risk prediction is possible with some remarkable softwares for Trisomy 18, Trisomy 13, Turner syndrome, triploidy, Cornela de Lange syndrome, Smith Lemlie Opitz syndrome and others. New markers like ADAM 12 with PaPPA will lead to early prediction of PIH and IUGR as well as prematurity. It is envisaged that in future maternal serum markers in combination with fetal biometry will give risk prediction for numerous chromosomal abnormalities many fetal disorders as well as pregnancy disturbances. Thus prenatal screening has an important place in antenatal care.

